# PiggyBac transposase-mediated inducible trophoblast-specific knockdown of *Mtor* decreases placental nutrient transport and fetal growth

**DOI:** 10.1042/CS20243293

**Published:** 2025-07-31

**Authors:** Fredrick J Rosario, Johann Urschitz, Haide Razavy, Marlee Elston, Theresa L Powell, Thomas Jansson

**Affiliations:** 1 Department of Obstetrics and Gynecology, University of Colorado, Anschutz Medical Campus, Aurora, CO, U.S.A.; 2 Institue of Biogenesis, University of Hawaii, Honolulu, HI, U.S.A.; 3 Section of Neonatology, Department of Pediatrics, University of Colorado Anschutz Medical Campus, Aurora, CO, U.S.A.

**Keywords:** placenta, maternal–fetal exchangeMaternal-Fetal Exchange, mechanistic target of rapamycin, fetal development, FGR

## Abstract

Abnormal fetal growth is associated with perinatal complications and adult disease. The placental mechanistic target of rapamycin (mTOR) signaling activity is positively correlated with placental nutrient transport and fetal growth. However, if this association represents a mechanistic link, it remains unknown. We hypothesized that trophoblast-specific *Mtor* knockdown in late pregnant mice decreases trophoblast nutrient transport and inhibits fetal growth. PiggyBac transposase-enhanced pronuclear injection was performed to generate transgenic mice containing a trophoblast-specific Cyp19I.1 promoter-driven, doxycycline-inducible luciferase reporter transgene with a *Mtor* shRNAmir sequence in its 3′ untranslated region (UTR). We induced *Mtor* knockdown by administration of doxycycline starting at E14.5. Dams were killed at E 17.5, and trophoblastspecific gene targeting was confirmed. Placental mTOR protein expression was reduced in these animals, which was associated with a marked inhibition of mTORC1 and mTORC2 signaling activity. Moreover, we observed a decreased expression of System A amino acid transporter isoform SNAT2 and the System L amino acid transporter isoform LAT1 in isolated trophoblast plasma membranes and lower fetal, placental weight, and fetal:placental weight ratio. We also silence the *MTOR* in cultured primary human trophoblast cells, which inhibited the mTORC1 and C2 signaling, System A and System L amino acid transport activity, and markedly decreased the trafficking of LAT1 and SNAT2 to the plasma membrane. Inhibition of trophoblast mTOR signaling in late pregnancy is mechanistically linked to decreased placental nutrient transport and reduced fetal growth. Modulating trophoblast mTOR signaling may represent a novel intervention in pregnancies with abnormal fetal growth.

## Introduction

Abnormal fetal growth, i.e., fetal growth restriction (FGR) or fetal overgrowth, is associated with increased perinatal morbidity and mortality and is strongly linked to the development of metabolic and cardiovascular disease in childhood and later in life [1–6]. Fetal growth is largely determined by oxygen and nutrient availability, controlled by placental blood flow and nutrient transport capacity [7,8]. Emerging evidence suggests that changes in placental amino acid transport expression and activity may contribute to abnormal fetal growth. Specifically, the activity of System A and L amino acid transporters has been reported to be decreased in human FGR [9–12]. In addition, various studies have reported an up-regulation of placental System A and L transport activity in fetal overgrowth [13–15].

The System L transporter is a sodium-independent exchanger mediating the transport of large neutral, predominantly essential amino acids, including leucine. System L is a heterodimer composed of a light chain, commonly known as LAT1 (*SLC7A5*) or LAT2 (*SLC7A8*), and a heavy chain, referred to as 4F2hc/CD98 (*SLC3A2*) [16]. Sodium-dependent neutral amino acid transporter 1 (SNAT1) (*SLC38A1*), SNAT2 (*SLC38A2*), and SNAT4 (*SLC38A4*) isoforms of the System A transporter are all expressed in the placenta [17] and preferentially mediate the active uptake of a range of neutral non-essential amino acids, including alanine, serine, and cysteine [18].

The mechanistic target of the rapamycin (mTOR) signaling pathway serves as a key regulator of many different cellular functions, ranging from cellular metabolism to growth and survival [19]. mTOR responds to various signals, such as hormones, growth factors, nutrients, energy availability, and stress signals [20,21]. mTOR is present in two distinct complexes: mTOR complex 1 (mTORC1) and mTORC2 [22,23]. The trophoblast mTOR pathway is considered a master regulator of placental function. For example, studies in cultured primary human trophoblast cells have demonstrated that mTOR signaling is a positive regulator of trophoblast amino acid (System A and L), folate transport, and mitochondrial respiration [24–26]. However, data unequivocally demonstrating that changes in trophoblast mTOR signaling regulate placental function *in vivo* are lacking.

Placental mTOR signaling has been proposed to serve as a critical hub for homeostatic control of fetal growth in response to maternal nutrition and metabolism perturbations, as well as uteroplacental blood flow [27,28]. In line with this model, we and others demonstrated that placental mTOR signaling is inhibited in FGR in women [12,29–32] and a range of animal models [33–41], while it is activated in fetal overgrowth [14,42–44]. Furthermore, placental mTOR signaling is reduced before the reductions in fetal growth, indicating that changes in the placental mTOR signaling could be the cause rather than a consequence of abnormal fetal growth [33]. Interestingly, inhibition of placental mTOR decreases the weight of female but not male fetuses [45]. However, compelling mechanistic evidence for the key role of trophoblast mTOR in regulating placental function and fetal growth is lacking.

Homozygous mTOR knockout embryos die shortly after implantation due to impaired cell proliferation in both embryonic and extraembryonic compartments [46], and maternal administration of the mTORC1 inhibitor rapamycin to pregnant mice results in FGR [47]. Hence, these results preclude defining the specific role of trophoblast mTOR signaling in regulating placental function and fetal growth. We hypothesized that trophoblast-specific *Mtor* knockdown in late pregnant mice decreases trophoblast nutrient transport and inhibits fetal growth. We employed *piggyBac* transposase-enhanced transgenesis [48] to achieve trophoblast-specific gene modulation. Specifically, PiggyBac transposase-enhanced pronuclear injection (te-PNI) was performed to generate transgenic mice by injecting vectors containing a trophoblast-specific Cyp19I.1 promoter-driven, doxycycline-inducible luciferase reporter transgene with mTOR shRNAmir sequence in its 3′ untranslated region (UTR), into one-cell B6D2F2 embryos. Here, we report that a reduction in trophoblast mTOR signaling is mechanistically linked to decreased trophoblast nutrient transport and reduced fetal growth. We propose that placenta-specific targeting of mTOR signaling represents a promising avenue to improve outcomes in pregnancies complicated by abnormal fetal growth. In addition, using gene silencing approaches in cultured primary human trophoblast cells, we tested the hypothesis that *MTOR* regulates trophoblast System A and L amino acid transporters isoform plasma membrane trafficking and System A and L amino acid transport activity.

## Materials and methods

### Animals and diet

Animals were housed out at the University of Hawaii, Honolulu, HI, United States. All animal work has taken place at the University of Hawaii, Honolulu, HI, United States. The Institutional Animal Care and Use Committee (Protocol # 13–1697) of the University of Hawaii approved all protocols conducted per NIH guidelines. All mice had *ad libitum* access to food and water.

### Construction of plasmid DNA

**Figure 1:**
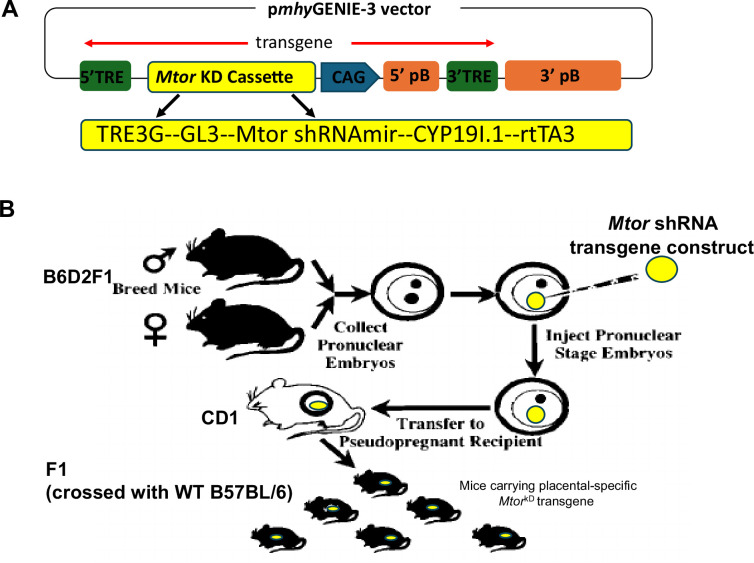
Transgene construct and experimental design for developing a trophoblast-specific inducible *Mtor* knockdown mouse. (**A**) A transgene construct is depicted, consisting of TRE3G & rtTA3, Tet-On system; GL3, luciferase reporter gene; shRNAmir, *Mtor* knockdown shRNAmir; CYP19I.1, Aromatase P450 fragment as the promoter for trophoblast-specific gene expression; CAG, CMV early enhancer/chicken beta-actin promoter; pB, piggyBac transposase and TRE, terminal repeat elements. (**B**) A schematic of the experimental design for developing a trophoblast-specific inducible *Mtor* knockdown mouse. Transposase-enhanced pronuclear injection was performed using B6D2F1 (B57BL/6 x DBA/2) and CD1 mice. Genomic DNA was isolated from pups, and genotyping PCR was performed to determine the number of transgenes for the founder generation and F1 (crossed with WT B57BL/6)

We used the Broad Institute’s Gene Perturbation website to identify five different shRNAs with *Mtor-*specific target sequences. Plasmids containing these shRNAs were obtained from Sigma (St. Louis, MO). We determined that the targeting sequence CCGTCCCTACATGGATGAAAT was most efficient in knocking down mTOR in primary human trophoblast cells. The basis for the final plasmid used for transgenesis was a commercially obtained TET-ON micro-RNA (miR30) adapted shRNA vector targeting the RNA of the mouse glucose transporter 1 (*Glut1,* V3THS_321626, Open Biosystems). This plasmid-encoded turboRFP under a TRE-3G promoter, with the reverse tetracycline-controlled transactivator 3 (rTTA3) driven by a ubiquitin C gene (UBC) promoter. The shRNAmir was located in the 3′ UTR of turboRFP. We replaced turboRFP with the luciferase reporter gene to enable *in vivo* assessment of transgene expression. The UBC promoter was replaced with the trophoblast-specific Cyp19I.1 promoter [49,50] to limit the luciferase and shRNAmir transgene expression to trophoblast cells. Finally, we exchanged the shRNA target sequence of *Glut1* with that of *Mtor*. These steps were all performed by restriction and insertion cloning. The transgene was then cloned into a pENTR1A vector (Thermo Fisher, Waltham, MA, U.S.A.) to facilitate the final step, the recombination of this *Mtor* knockdown pENTR1A vector with our pmhyGENIE-3 piggyBac vector [48,51], to generate the final construct (Figure 1a). The correct constructs were expanded, purified with gravity-flow columns, and used for transgenesis experiments.

### Development of a trophoblast-specific inducible mTOR knockdown mouse

te-PNI was performed [48,51–53] using B6D2F1 one-cell-stage embryos (B57BL/6 x DBA/2) and CD1 mice purchased from Jackson Laboratories (Figure 1b). Genomic DNA was isolated from pups, and genotyping PCR was performed. To determine the number of transgenes for the founder generation as well as F1 (crossed with WT B57BL/6), we performed transgene copy number assays by duplex Taqman real-time PCR, and results were analyzed using Applied Biosystems‘s Copy Caller software.

**Figure 2:**
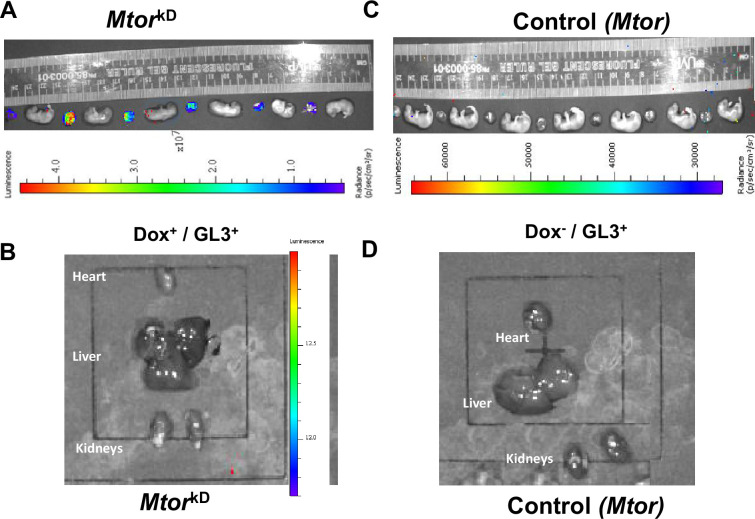
Trophoblast-specific inducible *Mtor* knockdown in mice. *Mtor* knockdown was induced by the administration of doxycycline at E14.5 in pregnant mice transgenic for a construct including a Tet-On system, luciferase, *Mtor* shRNA mir, and CYP19I.1. The vehicle was administered in the control transgenic animals (No Doxycycline). Laparotomy was performed at E17.5, and the luciferase signal was detected in isolated placentas and fetuses using an *in vivo* imaging system (IVIS). The luciferase signal could be detected only in the (**A**) placentas of dams that received doxycycline with no visible signal in the embryo proper, (**B**) maternal tissues, or (**C-D**) in transgenic animals in which *Mtor* knockdown had not been induced by doxycycline

Trophoblast-specific *Mtor* knockdown (*Mtor* ^kD^) was induced at E14.5 by administration of doxycycline 2.5 mg/kg (IP). Doxycycline injections were performed on the morning of E14.5. A total of 250 uL of a 5 mg/ml doxycycline solution (PBS) was injected intraperitoneally into each mouse. Doxycycline demonstrated no potential to cause toxicity (see below in results session). Transgenic animals in which vehicle was administered at E14.5 and wildtype females served as controls. At E17.5, there was no visible signal in transgenic animals in which *Mtor* knockdown had not been induced by doxycycline (Figure 2). In contrast, the luciferase signal could be detected in the placentas of dams that received doxycycline (Figure 2), with no visible signal in the embryo proper or maternal tissues.

### Immunofluorescence analysis for luciferase/cytokeratin/vimentin staining

Next, we demonstrated that our transgene construct specifically expresses in trophoblast cells and not in non-trophoblast cells. This was achieved by immunostaining placental sections of *Mtor* with anti-luciferase, anti-cytokeratin 7 (marker for trophoblast cells), and anti-vimentin (marker of non-trophoblast cells), as outlined below. In the current study, we aimed to restrict transgene expression specifically to the trophoblast cells of the placenta by utilizing the trophoblast-specific promoter, Cyp19I.1. This promoter is a 500  bp region located upstream of the CYP19 gene, which ensures that transgene expression is limited to trophoblast cells. Using the Cyp19I.1 promoter, we recently demonstrated that the expression of the *Deptor* gene decreased exclusively in the cytokeratin-7 positive cells, while it remained unaffected in the vimentin positive cells isolated from the placentas of transgenic mice [54].

Frozen sections of the placenta (7 μm) were fixed in methanol at –20°C for 20 minutes and washed in PBS. These sections were blocked in 10% normal goat serum diluted in antibody dilution buffer for 1 hour at room temperature. Mouse anti-luciferase (1:100; Thermo Scientific; MA1-16880), rabbit anti-vimentin (1:100; Cell Signaling 5741), and rabbit anti-cytokeratin 7 (1:50; Abcam AB181598) were added and incubated overnight at 4°C in a humidified chamber. Normal rabbit IgG (1:50; Thermo Scientific MA5-56524) and mouse IgG (1:100; Sigma-Aldrich; PP542) were substituted for primary antibody and served as a negative control. Tissue sections were then washed three times for 5 minutes per wash with PBS. Goat anti-rabbit IgG Alexa 488 and goat anti-mouse 594 IgG (Life Technologies; 1:250). Slides were counter stained with Prolong Gold Antifade reagent containing DAPI (4′,6-diamidino-2 phenylindole) (Life Technologies) and cover slipped. Images were taken using an Olympus CKX41 microscope.

Luciferase (red staining , Supplementary Figure S1a) expression was localized with cytokeratin-7-positive cells (green staining, Supplementary Figure S1a) but not with vimentin-positive cells (green staining, Supplementary Figure S1b). This suggests that our transgene construct is specific to only trophoblast cells in the placenta.

### Collection of placental tissue

Both control and experimental group dams were killed at E17.5 by carbon dioxide inhalation (Supplementary Figure S2). Following laparotomy, fetuses and placentas were collected and rapidly dried on blotting paper. After that, any remaining adherent fetal membranes and decidua were removed from fetuses and placentas before weighing. All placentas in each litter were pooled and washed briefly in PBS and transferred to 1 ml of ice-cold buffer D [250 mM sucrose, 10 mM HEPES-Tris, and 1 mM EDTA (pH 7.4) at 4°C]. A dilution of 1:1000 protease and phosphatase inhibitor cocktail (Sigma-Aldrich, St. Louis, MO) was added to the solution, which was then homogenized using a Polytron (Kinematica, Bohemia, NY). The mixture was frozen in liquid nitrogen and stored at −80°C until further processing and analysis. Small pieces of junctional zone (Jz) and labyrinth zone (Lz) were collected from randomly selected four placentas of each litter were pooled and flash frozen in liquid nitrogen and stored at –80°C until analysis.

### Gene expression analysis

RNA was extracted from frozen placental labyrinth/junctional zone and maternal/fetal tissues (liver, lung, heart, and brain) and reverse transcribed using commercially available kits (RNeasy Plus Mini kit, Qiagen and High-Capacity cDNA RT kit, Invitrogen, Carlsbad, CA). The expression of *Mtor* (Forward Sequence: GAGGAGCCTTCAGGATTACAAGATT; Reverse Sequence: GCTTTTGGCGAAGAAGGAGAATA) was determined by SYBR Green qRT-PCR using the relative standard curve method, relative to rRNA28S.

### Isolation of trophoblast plasma membranes

The trophoblast plasma membrane (TPM) is the maternal-facing plasma membrane of syncytiotrophoblast layer II of the mouse placentas, which is believed to be functionally similar to the syncytiotrophoblast microvillous plasma membrane of the human placenta [55]. As described elsewhere, TPMs were isolated from frozen placental homogenates using differential centrifugation and Mg^2+^ precipitation [55]. A Lowry assay (Bio-Rad, CA) was carried out to determine the protein concentration of TPM. The enrichment of TPM was assessed using the TPM/homogenate ratio of alkaline phosphatase activity. There was no significant difference in the average enrichment of alkaline phosphatase in TPM vesicles isolated from *Mtor* knockdown animals (13.6 ± 0.6, *n* = 6) as compared with placentas of transgenic animals injected with vehicle (14.4 ± 0.8, *n* = 7) or placentas of wildtype animals (14.0 ± 0.9, *n* = 7).

### TPM amino acid transporter activity measurements

The TPM System A and L amino acid transporter activity was measured using radiolabeled amino acids and rapid filtration techniques [36,55]. TPM vehicles from WT, *Mtor*, and *Mtor* ^kD^ groups were preloaded by incubation in 300 mM mannitol and 10 mM Hepes-Tris, pH 7.4 overnight at 4°C. Subsequently, TPM vesicles were resuspended in a small volume of the same buffer after being pelleted (final protein concentration: 5–10 mg ml^-1^). Membrane vesicles were kept on ice until transport activity measurements were performed. Immediately before transport activity measurements, TPM vesicles were warmed to 37°C. At time zero, 30 μl of TPM vesicles was quickly mixed (1:2) with the incubation buffer containing [^14^C] methyl-aminoisobutyric acid (MeAIB, 150 μM) with or without Na^+^ or L-[^3^H] leucine (0.375 μM). Based on previous time course studies [36], uptake at 15 seconds was used in all subsequent experiments. The uptake of radiolabeled substrate was terminated by adding 2 ml of ice-cold PBS. Subsequently, vesicles were rapidly separated from the substrate medium by filtration on mixed ester filters (0.45 μm pore size, Millipore Corporation, Bedford, MA, U.S.A.) and washed with 3 × 2 ml of PBS. Each condition was studied in duplicate for each membrane vesicle preparation in all uptake experiments. Filters were dissolved in 2 ml of liquid scintillation fluid (Filter Count, PerkinElmer, Waltham, MA, U.S.A.) and counted. Appropriate blanks were subtracted from counts and uptakes expressed as pmol (mg protein) ^−1^. Na^+^-dependent uptake of MeAIB (corresponding to System A activity) was calculated by subtracting Na^+^-independent from total uptakes. Mediated uptake was calculated for leucine by subtracting non-mediated transport, as determined in the presence of 20 mM unlabeled leucine, from total uptake.

### Human subjects

Placentas from uncomplicated term C-section deliveries were collected to isolate cytotrophoblast cells, which were then cultured *in vitro*. The placental collection was authorized by the University of Colorado Anschutz Medical Campus Institutional Review Board (COMIRB 14–1073). Every person who was recruited gave written informed consent, and the study protocol was carried out in compliance with the World Medical Association Declaration of Helsinki. Smoking, illegal drug use, co-occurring conditions like diabetes and hypertension, the onset of pregnancy complications like gestational diabetes, pregnancy-induced hypertension, and preeclampsia, as well as fetal abnormalities, preterm birth, and birth-related complications, were all considered exclusion criteria. Selected clinical data presented in Table 1.

**Table 1:** Selected clinical data^a^.

Maternal age (years)	29.5 ± 2.5
BMI (kg/m^2^)	20.6 ± 0.12
Gestational age (weeks)	40.0 ± 0.40
Birth weight (g)	3352 ± 232
Birth weight percentileb	35.3 ± 6.8
Placental weight (g)	638 ± 24
Fetal sex (M/F)	4/2
Mode of delivery (C/V)	(6/0)

Data are presented as means ± S.E.M.

a Data from n=6 Appropriate for gestational age pregnancy

b By corresponding gestational age.

F, female. M, male. C, caesarean section. V, vaginal delivery.

### Cytotrophoblast isolation and culture from term placentas

After the collection of the placenta, primary human trophoblast (PHT) cells were isolated utilizing an established method that included sequential trypsin digestion and Percoll purification [26]. Following isolation, cells were cultured in Dulbecco’s Modified Eagle’s Medium (DMEM, Sigma-Aldrich, St. Louis, MO) and Ham’s F-12 nutrient mixture (Life Technologies, Carlsbad, CA), supplemented with 10% fetal bovine serum (FBS, Atlanta Biological, Atlanta, GA), 50 μg/ml gentamicin, 60 μg/ml benzyl penicillin, and 100 μg/ml streptomycin (Sigma-Aldrich). Cells were cultured in either 60 mm culture dishes (approximately 7.5 × 10^6^ cells/dish for Western blot analysis) or six-well plates (approximately 2.75 × 10^6^ cells/well for amino acid uptake) and incubated in a 5% CO_2_, 95% atmosphere air at 37°C for 90 hours, with daily media replacement. All experiments were replicated in PHT cells isolated from six different placentas.

### RNA interference-mediated silencing of *MTOR*

Dharmafect 2 transfection reagent (Thermo Scientific) and siRNA (SignalSilence® *MTOR* siRNA I, Cat #6381), targeting *MTOR* (100 nM), were used. Control cells were transfected with a non-coding scrambled sequence (100 nM; sense: 5′GAUCA-UACGUGCGAUCAGATT). siRNAs were added to cultured PHT cells after 18 h in culture, incubated for 24 h and subsequently removed and replaced by fresh medium [26]. At 90 h in culture, efficiency of target silencing was determined at the protein and functional levels using Western blot.

### System A and System L amino acid uptake assay

Next, we determined the System A and L amino acid uptake of primary human trophoblast cells at 90 h in culture. The uptake activity of System A and System L amino acid transporters was assessed by determining the Na^+^-dependent uptake of [^14^C] methyl-aminoisobutyric acid (MeAIB; 20 μM) and the 2-amino-2-norbornane-carboxylic acid (BCH; 64 µM)-inhibitable uptake of [^3^H] leucine (0.0125 μM), as previously described [26].

### Isolation of microvillous plasma membrane from PHT cells

Microvillous plasma membranes (MVMs) were isolated from whole cell lysates of cultured PHT cells, as previously mentioned [26]. Briefly, PHT cells were lysed, homogenized, and centrifuged. The pelleted crude cell membrane fraction was resuspended and added with 12 mM MgCl_2_ for membrane precipitation. The cell membrane fraction mixture was stirred at a slow speed for 20 minutes on ice and subsequently centrifuged. The supernatant containing MVM was subjected to centrifugation at 125,000 ***g*** for 30 minutes, and the resultant pellet was resuspended. The Bradford assay was employed to determine protein concentration. Using the MVM/homogenate ratio of alkaline phosphatase activity, MVM enrichment was evaluated. There was no significant difference in the average enrichment of alkaline phosphatase in MVM isolated from PHT cells transfected with either scramble siRNA (Control, 9.4 ± 0.93, *n* = 5/group) or *MTOR* (9.8 ± 1.1, *n* = 5/group) siRNA.

### Isolation of fetal facing basal plasma membranes from PHT cells

Basal plasma membranes (BMs) were isolated from cultured PHT cells based on a previously described method [24]. The enrichment of voltage-dependent anion channel (VDAC) [56], a BM marker, was determined by the ratio of VDAC expression in BM to total cell lysates. The VDAC enrichment in BM isolated from PHT cells transfected with scramble siRNA (Control, 5.7 ± 0.60, *n* = 5/group), which was not different from BM isolated from or *MTOR* siRNA (Control, 6.0 ± 0.72, *n* = 5/group) silenced cells.

### Western blot analysis

Western blot analysis was carried out as described [14,26,33,36]. Briefly, 10–15 μg of placental homogenates or 5–10 μg of TPM was loaded onto a NuPAGE Bis-Tris Gels (Thermo Fisher Scientific), and electrophoresis was performed using MOPS-SDS running buffer at a constant 100 V for 1 h. After gel electrophoresis, the separated proteins were transferred onto nitrocellulose membranes using NuPAGE™ Transfer Buffer (Invitrogen) at a constant 30 V for 1 h. After transfer, nitrocellulose membranes were blocked in 5% blotting grade blocker nonfat dry milk (Bio-Rad, Hercules, CA) in TBS (wt/vol) plus 0.1% Tween 20 (vol/vol) for overnight at 4°C. Following serial washing in TBS plus 0.1% Tween 20 for 1 hour at room temperature, the membrane was incubated with appropriate primary antibody overnight at 4°C. Subsequently, the membranes were washed in TBS plus 0.1% Tween 20 for 1 h at room temperature, followed by incubation for 1 hour with the corresponding secondary peroxidase-labeled antibodies. Following the washing step in TBS plus 0.1% Tween 20, the bands were visualized using enhanced chemiluminescence detection reagents from Thermo Fisher Scientific (Waltham, MA, U.S.A.). Blots were stripped [14,33,36] and reprobed for β-actin. Densitometry analysis of target protein bands of the immunoblots was performed with Gene Tools (SynGene). The mean density of the control sample bands was given a value of 1 for each protein target, and the data is presented in relation to the control. The relative density of the target protein in each lane was normalized by dividing it by the density of the corresponding total protein or β-actin band, which served as a loading control. Additionally, no significant difference was observed in β-actin expression between the control and experimental groups (data not presented). Total protein staining of Western blot membrane was performed using Pierce™ Reversible Protein Stain Kit (Thermo Fisher Scientific, Waltham, MA).

Isolated TPM (isolated from mice placental homogenate) or MVM (isolated from PHT cells) or BM (isolated from PHT cells) was used to measure the protein expression of System A amino acid transporter isoform (SNAT) 2 (*SLC38A2*) and the System L amino acid transporter isoform LAT1(*SLC7A5*). A polyclonal SNAT2 antibody generated in rabbits was received as a generous gift from Dr. V. Ganapathy and Dr. P. Prasad at the University of Georgia, Augusta. Antibodies targeting the LAT1 were produced in rabbits and received as a generous gift from Dr Yoshikatsu Kanai from Osaka University, Osaka, Japan. The specificity of SNAT2 and LAT1 antibodies has previously been validated using gene-silencing techniques [57,58]. Furthermore, mTOR signaling pathway downstream signaling activity in placental homogenates was measured by Western blot for total and phosphorylated forms of mTOR, S6 (Ser-235/236), 4EBP1 (Thr-70), and Akt (Ser-473). In addition, full-length PARP, caspase-3, and cleaved caspase-3 expression in placental homogenate was measured by Western blot. Protein expression of mTOR and phosphorylated forms of S6 (Ser-235/236), Akt (Ser-473) in PHT cell lysate was measured by Western blot.

### Data presentation and statistics

GraphPad Prism 10.4.0 software was used to analysis data.

For animal experiment: Data are presented as mean ± SEM. All placentas in a litter of the respective genotypes (WT, *Mtor*, and *Mtor* ^kD^) were pooled prior to the molecular analysis and transport assays. Thus, *n* represents the number of litters. The distribution of data was assessed by the D’Agostino & Pearson test and Shapiro–Wilk test. When data showed normal distribution (WT *vs Mtor vs Mtor^kD^*), statistical significance between WT, *Mtor*, and *Mtor* ^kD^ was determined by one-way ANOVA with Tukey–Kramer multiple comparisons post hoc test (*P*<0.05). When data were not normally distributed, it was analyzed Kruskal–Wallis test with Dunn’s multiple comparisons test.

For PHT cell experiment: Data are presented as mean ± SEM. The number of experiments (*n*) represents the number of placentas studied. In the uptake (amino acid) experiments, each condition was studied in triplicate, and data were averaged to represent trophoblast cells isolated from one placenta. The distribution of data was assessed by the D’Agostino & Pearson test and Shapiro–Wilk test. Data from control and *MTOR* siRNA were normally distributed. The statistical significance of differences between *MTOR* siRNA and control (scramble) group was assessed using Student’s paired *t*-test. A P value < 0.05 was considered significant.

## Results

### Doxycycline treatment did not induce toxicity in mice

**Figure 3:**
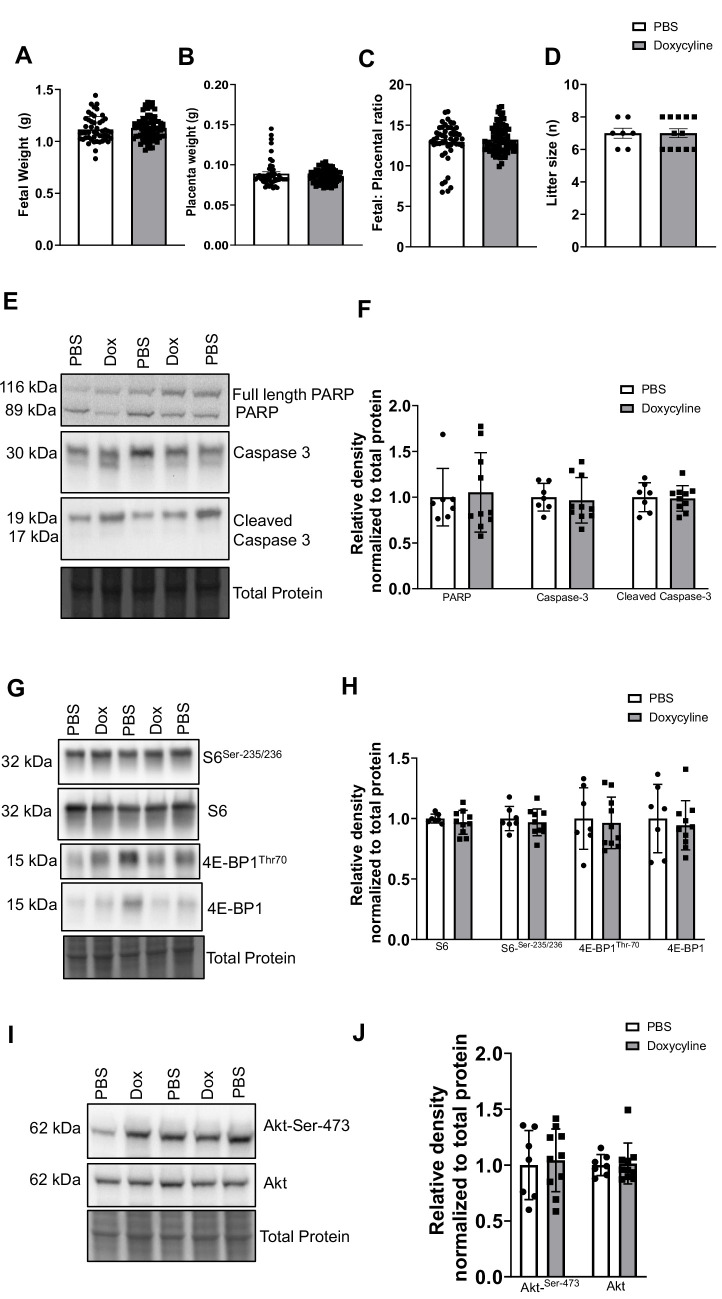
Doxycycline treatment did not affect fetal and placental weight and placental caspase/mTOR signaling in mice. (**A-D**) Doxycycline (2.5 mg/kg (IP)) was administered at E14.5 in pregnant wild type (WT) mice. The vehicle (PBS) was administered to pregnant WT mice (No Doxycycline, Control). At E18.5, animals were sacrificed, and fetal and placentas from each litter were weighed individually. Fetal weight (**A**), placental weight (**B**), fetal: placental weight ratio (**C**) and litter size (**D**) in control and doxycycline administered conceptuses (PBS group: *n* = 7 dams, 49 conceptuses; Doxycycline group: *n* = 11 dams, 78 conceptuses). (**E-J**) Doxycycline treatment did not affect placental PARP (Poly (ADP-ribose) polymerase), caspase 3/cleaved caspase 3, mTORC1 and mTORC2 signaling in mice. Placentas from each litter were pooled and homogenized. Western blot determined the (**E,F**) PARP (analyzing 89 and 116 kDa bands together)/caspase 3/cleaved caspase 3, total expression and phosphorylation of key intermediates in the (**G,H**) mTORC1 (S6^Ser-235/236^, total S6, 4E-BP1 ^Thr-70^, and 4E-BP1) and (**I,J**) mTORC2 (Akt^ser-473^ and total Akt) signaling pathways. (**E,G,I**) Representative western blots of PARP/caspase 3/cleaved caspase 3, S6^Ser-235/236^, total S6, 4E-BP1 ^Thr-70^, 4E-BP1, Akt^ser-473^ and total akt expression in placental homogenates of vehicle (PBS) and doxycycline administered mice. Equal loading was performed. (**F,H,I**) Summary of the western blot data. *n* = 7–10 dams in each group. Values are expressed as means ± SEM

Here, we demonstrated that administration of doxycycline (2.5 mg/kg (IP) at E14.5) did not affect the placental and fetal weights. As shown in Figure 3a–c, administration of doxycycline did not affect the fetal (*P*=0.541) and placental weight (*P*=0.206) and fetal:placental weight ratio (*P*=0.425, *n* = 7–11 dams/group and *n* = 49–78 conceptuses/group) in WT as compared with WT administered with PBS (Control). Furthermore, litter size (*P*=0.999, *n* = 7–11 dams/group) was comparable between groups (Figure 3d). In addition, the protein expression of poly (ADP-ribose) polymerase (PARP- a marker of DNA damage; *P*=0.788, *n* = 7–10 dams/group, analyzing 89 and 116 kDa bands together), caspase 3 (*P*=0.753, *n* = 7–10 dams/group), and cleaved caspase 3 (*P*=0.865, *n* = 7–10 dams/group) in the placenta was comparable between doxycycline injected and control group (Figure 3e and f). Furthermore, both mTOC1 (S6^Ser-235/236^: *P*=0.422, S6: *P*=0.558, 4E-BP1^Thr-70^: *P*=0.756, 4E-BP1: *P*=0.641, *n* = 7–10 dams/group, Figure 3g and h) and mTORC2 (Akt^Ser-473^: *P*=0.769, Akt: *P*=0.858, *n* = 7–10 dams/group, Figure 3i and j) downstream target protein expressions were comparable between groups, suggesting that doxycycline did not affect fetal, placental weight, and placental apoptosis/mTOR signaling pathways in the mice.

### Trophoblast-specific inducible *Mtor* knockdown decreases placental mTOR expression in mice

**Figure 4:**
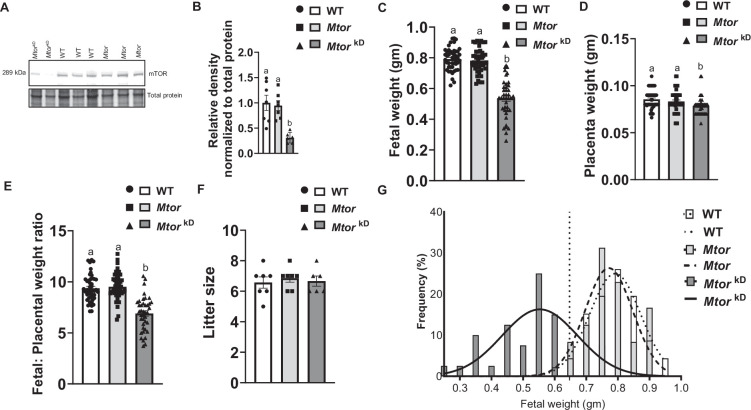
Trophoblast-specific inducible *Mtor* knockdown in mice decreases placental mTOR protein expression and fetal weight. Trophoblast**-**specific *Mtor* knockdown was induced by the administration of doxycycline starting at E14.5 (*Mtor* ^kD^). The vehicle was administered at E14.5 in control animals, resulting in mice without *Mtor* knockdown (*Mtor*). At E17.5, animals were sacrificed, and placentas from each litter were collected, pooled and homogenized. Western blot was used to determine the total mTOR protein expression. (**A**) Representative western blot of total mTOR expression in placental homogenates of WT, *Mtor* and *Mtor* ^kD^ placentas. Equal loading was performed. (**B**) Summary of the western blot data. *n* = 6–7 dams in each group. (**C-G**) Fetal and placental weight, fetal: placental (**F:P**) weight ratio, litter size and fetal weight distribution curves following induction of trophoblast-specific *Mtor* knockdown in mice. at E17.5, animals were sacrificed, and placentas from each litter were weighed. Compared with control and wildtype mice, trophoblast-specific *Mtor* knockdown (induced by doxycycline at E14.5) mice exhibit reduced (**C**) fetal weights and (**D**) placental weights at embryonic day 17.5. Trophoblast-specific *Mtor* knockdown in mice reduced (**E**) fetal:placental weight ratio but not (**F**) litter size. (**C-F**) Symbols show the individual fetal/ placental weight, and F:P ratio in WT, *Mtor,* and *Mtor* ^kD^ dams. Values are expressed as means ± SEM. (**B,C,E**) Means without a common letter are statistically different by one-way ANOVA with Tukey–Kramer multiple comparisons post hoc test (*P*<0.05). Each value represents individual fetal weight, placental weight, and F:P ratio in WT (*n* = 46 fetuses from 7 dams), *Mtor* (*n* = 48 fetuses from 7 dams)*,* and *Mtor* ^kD^ (*n* = 40 fetuses from 6 dams) dams. (**D**) Means without a common letter are statistically different by Kruskal–Wallis test with Dunn’s multiple comparisons test (*P*<0.05). (**F**) Frequency distribution of individual fetal weights in WT (*n* = 46), *Mtor* (*n* = 48)*,* and *Mtor* ^kD^ (*n* = 40) dams. Mean fetal weight of trophoblast-specific *Mtor* knockdown mice (solid line, *r* ^2^ = 0.67; *n* = 40 fetuses, 6 dams) was significantly lower than in wildtype (WT, dotted line, *r* ^2^ = 0.99; *n* = 46 fetuses, 7 dams) and control type (*Mtor*, dashed line, *r* ^2^ = 0.84; *N* = 48 fetuses, 7 dams) mice. The vertical dashed line represents the 5th centile on the WT curve (0.647 gm), revealing 77.5% of *Mtor* ^kD^ fetuses fall below this. Values are expressed as means ± SEM

Placental mTOR protein expression was 69% (*P*=0.0008, *n* = 6–7 dams/each group) lower in the *Mtor* ^kD^ group than in the WT and *Mtor* (Control) groups (Figure 4a and b). Similarly, when placentas were dissected to separate the nutrient-transporting labyrinth zone from the hormone-producing junctional zone, *Mtor* knockdown reduced *Mtor* mRNA expression in both labyrinth (−62%, *P*=0.0001, *n* = 6–7 dams/group) and junctional zones (−70%, *P*=0.0001, *n* = 6–7 dams/group) as compared with control group ( Supplementary Figure S3 a-b). However, *Mtor* mRNA expression in non-placental tissues did not differ between WT and *Mtor* (Control) and *Mtor* ^kD^ group, in either the maternal/fetal liver or lung or brain or heart (Supplementary Figure S4 a-h) or decidua or uterus or fetal membrane (Supplementary Figure S5 a-c), consistent with trophoblast-specific knockdown of the *Mtor* gene.

### Trophoblast-specific inducible *Mtor* knockout decreases fetal and placenta weight in mice

As shown in Figure 4c, Supplementary Figure S6a, trophoblast-specific inducible *Mtor* knockout decreases fetal weight (−32 %, *P*=0.005, *n* = 6–7 dams/each group and 40–48 conceptuses/each group) as compared with WT and *Mtor* (Control) groups. In addition, placenta weight was lowered by 8% (*P*=0.002, *n* = 6–7 dams/each group and 40–48 conceptuses/each group) in *Mtor* knockout mice as compared with WT, and *Mtor* (Control) groups (Figure 4d, Supplementary Figure S6b). Placental and fetal weight ratio was significantly (*P*=0.001, *n* = 6–7 dams/each group and 40–48 conceptuses/each group) decreased in *Mtor* knockout mice as compared with WT, and *Mtor* (Control) groups (Figure 4e, Supplementary Figure S6c). Litter size was comparable between groups (Figure 4f). To characterize the observed FGR in more detail, fetal weight distribution curves were constructed for WT, *Mtor,* and *Mtor* ^kD^ groups (Figure 4g). Fetal weight distribution of *Mtor* ^kD^ fetuses was shifted to the left (indicative of a lower weight) and 77.5% of the *Mtor* ^kD^ fetuses had a weight below the 5th centile of the normal WT distribution. No fetal demise was observed in control or experimental groups.

### Trophoblast-specific inducible *Mtor* knockdown decreases placental mTORC1 signaling in mice

**Figure 5:**
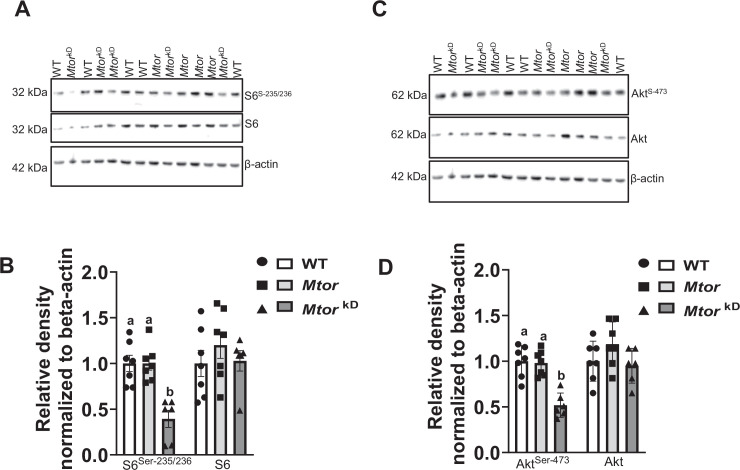
Placental mTORC1 and mTORC2 signaling expression following induction of trophoblast-specific *Mtor* knockdown in mice. Trophoblast-specific *Mtor* knockdown was induced by the administration of doxycycline starting at E14.5 (*Mtor* ^kD^). The vehicle was administered at E14.5 in control animals, resulting in mice without *Mtor* knockdown (*Mtor*). At E17.5, animals were sacrificed, and placentas from each litter were weighed, pooled and homogenized. (**A-B**) Inhibition of placental mTORC1 signaling following induction of trophoblast-specific *Mtor* knockdown. Western blot determined the total expression and phosphorylation of key intermediates in the mTORC1 signaling pathways. (**A**) Representative western blots of S6^Serine-235/236^, and total S6 expression in placental homogenates of WT, *Mtor* and *Mtor* ^kD^ placentas. (**C-D**) Inhibition of placental mTORC2 signaling following induction of trophoblast-specific *Mtor* knockdown. (**C**) Representative western blots of Akt ^Serine-473^, and Akt expression in placental homogenates of WT, *Mtor,* and *Mtor* ^kD^ placentas. Equal loading was performed. (**B,D**) Summary of the western blot data. *n* = 6–7 dams in each group. Values are expressed as means ± SEM. (**B**) Means without a common letter are statistically different by Kruskal-Wallis test with Dunn’s multiple comparisons test (*P*<0.05). (**D**) means without a common letter are statistically different by one-way ANOVA with Tukey–Kramer multiple comparisons post hoc test (*P*<0.05).

To further explore the mechanisms linking trophoblast-specific inducible *Mtor* knockdown to decreased fetal growth, we used Western blot to determine functional readouts of the *Mtor* signaling pathways in WT, *Mtor*, and *Mtor* ^kD^ placentas. Trophoblast-specific *Mtor* knockdown significantly decreased 61% (*P*=0.0004, *n* = 6–7 dams/each group) the S6 ribosomal protein phosphorylation at Serine 235/236, a mTORC1 downstream target. Total S6 ribosomal protein expression level was comparable between groups (Figure 5a–b).

### Trophoblast-specific inducible *Mtor* knockdown decreases placental mTORC2 signaling in mice

Trophoblast-specific *Mtor* knockdown significantly decreased the phosphorylation of protein kinase B (Akt-Ser 473; -49%, *P*=0.0001, *n* = 6–7 dams/each group), functional readouts of mTORC2 activity, as compared with WT and *Mtor* (Control) group at E 17.5 (Figure 5c–d). Moreover, the total expression of Akt was not different in WT, *Mtor*, and *Mtor* ^kD^ groups.

### Trophoblast-specific inducible *Mtor* knockdown decreases placental TPM SNAT2 and LAT1 expression

**Figure 6:**
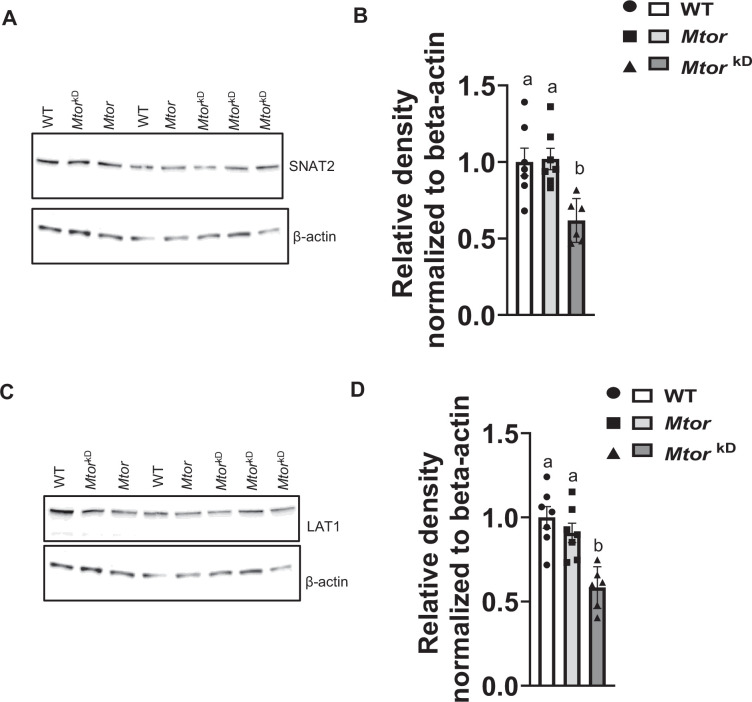
Placental trophoblast plasma membrane SNAT2 and LAT1 expression following induction of trophoblast-specific *Mtor* knockdown in mice. Trophoblast-specific *Mtor* knockdown was induced by the administration of doxycycline starting at E14.5 (*Mtor* ^kD^). The vehicle was administered at E14.5 in control animals, resulting in mice without *Mtor* knockdown (*Mtor*). At E17.5, animals were sacrificed, and placentas from each litter were weighed, pooled and homogenized. (**A-B**) Decreased trophoblast plasma membrane SNAT2 and LAT1 expression following induction of trophoblast-specific *Mtor* knockdown. trophoblast plasma membranes were isolated, and the protein expression of the amino acid transporter isoforms SNAT2 (System A) and LAT1 (System L) was determined using Western blot. (**A,C**) Representative western blots of SNAT2 and LAT1 expression in TPM of WT, *Mtor,* and *Mtor* ^kD^ placentas. Equal loading was performed. (**B,D**) Summary of the Western blot data. *n* = 6–7 dams in each group. Values are expressed as means ± SEM. means without a common letter are statistically different by one-way ANOVA with Tukey–Kramer multiple comparisons post hoc test (*P*<0.05). .

To mediate cellular uptake and transplacental transport, the SNAT2 and LAT1 proteins must be translocated to the trophoblast plasma membrane. In addition, membrane trafficking of SNAT2 and LAT1 is tightly regulated in cultured primary human trophoblast cells by mTOR signaling [26]. We used Western blotting to determine SNAT2 and LAT1 protein abundance in TPM isolated from WT, *Mtor*, and *Mtor* ^kD^ placentas. As shown in Figure 6a–d, TPM SNAT2 (−49%, *P*=0.003, *n* = 6–7 dams/each group) and LAT1 (−42 %, *P*=0.0003, *n* = 6–7 dams/each group) were significantly lower in the *Mtor* ^kD^ group as compared with WT and *Mtor* groups.

## Trophoblast-specific inducible *Mtor* knockdown decreases placental TPM System L and System A amino acid transport

**Figure 7:**
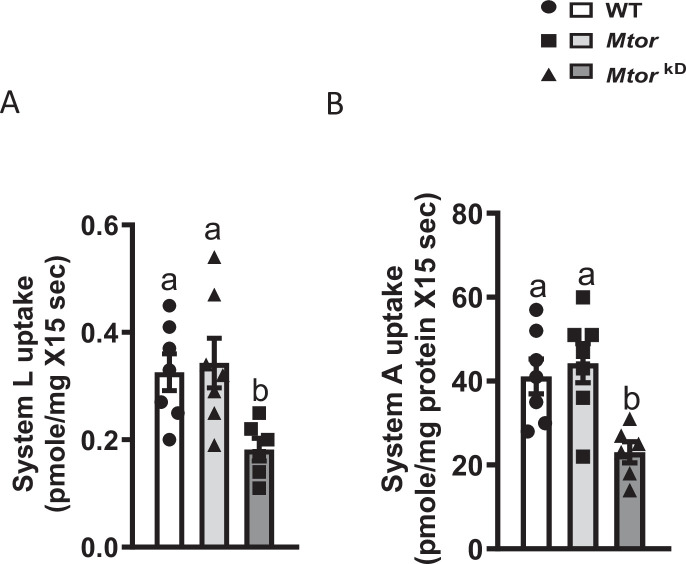
Decreased trophoblast plasma membrane System L and System A activities following induction of trophoblast-specific *Mtor* knockdown. *Mtor* knockdown was induced by the administration of doxycycline starting at E14.5 (*Mtor* ^kD^). In control transgenic animals (*Mtor*), the vehicle was administered at E14.5. At E17.5, animals were sacrificed, and placentas from each litter were pooled and homogenized. Trophoblast plasma membranes were isolated. System L (**A**) and System A (**B**) transporter activities were determined using isotope-labeled substrates and rapid filtration techniques in TPM isolated from WT, *Mtor,* and *Mtor* ^kD^ placenta at E 17.5. Values are expressed as means ± SEM. means without a common letter are statistically different by one-way ANOVA with Tukey–Kramer multiple comparisons post hoc test (*P*<0.05). .

In TPM isolated from *Mtor* ^kD^ placentas, the capacity to transport System L (−45%, *P*=0.01, *n* = 6–7 dams/each group) and System A (−44%, *P*=0.004, *n* = 6–7 dams/each group) amino acid transport was markedly decreased compared with TPM isolated from WT, or *Mtor* placentas (Figure 7a and b).

### *MTOR* silencing decreases the mTORC1 and mTORC2 signaling in PHT cells

We silenced the *MTOR* expression in cultured primary human trophoblast cells and measured the protein expression of functional readouts of mTORC1 and mTORC2 signaling. As shown in Supplementary Figure S7 a-b, *MTOR* siRNA decreased the mTOR protein expression by 55% (*P*=0.002, *n* = 6/group) as compared with scramble. In addition, *MTOR* silencing in PHT cells significantly decreased 50% (*P*=0.03, *n* = 5/group) the S6 ribosomal protein phosphorylation at Serine 235/236, a mTORC1 downstream target (Supplementary Figure S8 a-b). Furthermore, *MTOR* silencing decreased 48% (*P*=0.005, *n* = 5/group) the phosphorylation of Akt (Serine-473, mTORC2 downstream target) as compared with the scramble ( Supplementary Figure S9 a-b).

#### *MTOR* silencing decreases the PHT System L and A amino acid transport activity and MVM LAT1/SNAT2 isoform expression

Silencing of *MTOR* in PHT cells decreased System L (−54%. *P*=0.0007, *n* = 6/group) and System A amino acid transport (−43%, *P*=0.001, *n* = 6/group) as compared with PHT cells transfected with scramble sequences (Supplementary Figure S10 a-b). Furthermore, we found that *MTOR* silencing decreased the expression of the System L amino acid transporter isoform LAT1 (−68%, *P*=0.03, *n* = 5/group,Supplementary Figure S11a,b) and System A amino acid transporter isoform SNAT2 (−54%, *P*=0.002, *n* = 5/group, Supplementary Figure S11a, c) in the microvillus plasma membranes (maternal facing plasma membrane) isolated from cultured PHT cells. This suggests that *MTOR* silencing decreases the trafficking of LAT1 and SNAT2 to the MVM of PHT cells.

### *MTOR* silencing decreases the trafficking of LAT1 to the basal plasma membrane of PHT cells

Subsequently, we examined the effect of *MTOR* silencing on expression of basal plasma membrane (fetal facing plasma membrane) System L amino acid transporter isoform LAT1 in cultured PHT cells. MTOR silencing resulted in a notable reduction in the expression of the System L transporter isoform LAT1 in the BM fraction (-44%, *P*=0.0002, *n* = 5/group, Supplementary Figure S12 a-b). These data demonstrate that MTOR modulates the expression of the BM System L amino acid transporter isoform in trophoblast cells.

## Discussion

**Figure 8:**
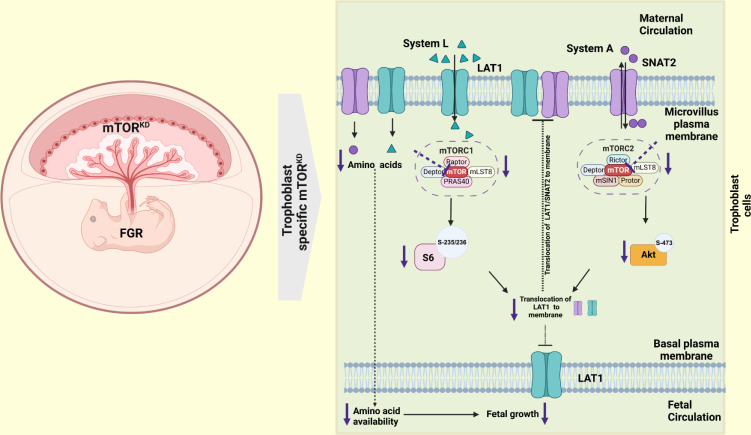
The present study’s findings are consistent with the model that trophoblast-specific knockdown of *Mtor* mRNA in mice is mechanistically linked to decreased trophoblast mTORC1 and mTORC2 signaling. Inhibition of placental mTORC1 and mTORC2 signaling decreased the transporter trafficking of System L amino acid transport isoform LAT1 and System A amino acid transport isoform SNAT2, which contributes to decreased fetal amino acid supply and fetal growth restriction.

Our study represents the first application of piggyBac transposase-enhanced transgenesis to achieve inducible trophoblast-specific gene modulation. In addition, we demonstrate that the inhibition of trophoblast mTOR signaling is mechanistically linked to decreased placental nutrient transport and reduced fetal growth (Figure 8). In addition, we showed that silencing of *MTOR* expression in PHT cells decreased the System A and System L amino acid transport activity by decreasing the microvillus plasma membrane expression of specific System A (SNAT2, *SLC38A2*) and System L (LAT1, *SLC7A5*) isoforms. Furthermore, this study demonstrates that *MTOR* regulates the fetal-facing basal plasma membrane trafficking of LAT1 in PHT cells.

Despite their introduction to the field many years ago, the successful use of approaches to achieve trophoblast-specific gene modulation has been rather limited. For example, the hCYP promoter-Cre construct is associated with some degree of ‘leakiness’, sometimes resulting in expression of the construct in fetal tissues such as the brain, eye, skin and heart [59]. Moreover, placental defects are highly prevalent in embryonic lethal mouse mutants [60], including *Mtor,* precluding meaningful studies of the function of some critical placental genes in the second half of pregnancy. Herein, we report using piggyBac transposase-enhanced transgenesis to achieve trophoblast-specific gene knockdown. We believe that our approach represents a robust tool for inducible trophoblast-specific gene modulation that will allow the placental research field to conduct detailed mechanistic studies of the function of hundreds of placental genes *in vivo* without risking that global deletion of these genes results in embryonic lethality. In addition, many placental genes regulate both placental development and the function of the mature placenta, requiring experimental models with temporal control of trophoblast-specific gene modulation, such as piggyBac transposase-enhanced transgenesis, to differentiate between these two distinct processes.

We observed no toxicity of doxycycline given intraperitoneal injection (2.5 mg/kg) to pregnant mice on E14.5, which did not induce placental apoptosis and mTOR signaling. Taken together these data suggest that doxycycline concentration used in the current study did not affect placental and fetal weights. Placental mTORC1 and 2 signaling pathways are positive regulators of key amino acid transporters in PHT cells and human placental villous explants [26,29,61]. Inhibition of mTORC1 and/or mTORC2 down-regulates trophoblast System A and L amino acid transporter activity by decreasing the plasma membrane expression of specific System A (SNAT2, *SLC38A2*) and System L (LAT1, *SLC7A5*) isoforms with no effect on overall protein expression [34]. Distinct mechanisms mediate the regulation of SNAT2 and LAT1 membrane trafficking by mTORC1 and 2. Inhibition of mTORC1 results in activation of Nedd 4–2, an E3 ubiquitin ligase, which ubiquitinates the specific amino acid transporter isoforms leading to their withdrawal from the plasma membrane and degradation [25]. In contrast, inhibition of mTORC2 decreases SNAT2 and LAT1 plasma membrane abundance in PHT cells mediated by down-regulation of Rho GTPase Cdc42 and Rac1 [24], which are essential for actin skeleton function. In the current study, trophoblast-specific knock-down of *Mtor*, resulting in a marked inhibition of placental mTORC1 and mTORC2 signaling, decreased the protein expression of SNAT2 and LAT 1 in the trophoblast plasma membrane, providing the first in vivo evidence of mTOR controlling the trophoblast plasma membrane trafficking of these two specific amino acid transporter isoforms. Importantly, using isolated trophoblast plasma membrane vesicles incubated with radiolabeled amino acids and rapid filtration, we confirmed that trophoblast-specific mTOR knockdown causes a marked decrease in System A and L amino acid transporter function. We propose that a decreased transplacental transport of amino acids causes the inhibition of fetal growth following trophoblast-specific mTOR knockdown in our model. This aligns with our recent study demonstrating that placental-specific Slc38a2 knockdown in mice resulted in FGR [58].

Fetal weight was reduced in trophoblast-specific inducible *Mtor* knockout mice, which is similar in magnitude to that observed in other mouse models either with inhibition of placental mTOR signaling due to maternal folate deficiency [37], or following administration of rapamycin to pregnant mice [62]. Our study demonstrates that the inhibition of mTOR signaling only in the trophoblast is sufficient to cause FGR. Highlighting the translational relevance of these findings, previous studies have demonstrated that placental mTOR protein expression and/or mTOR signaling activity is down-regulated in human FGR [30,31,62]. Using Trophoblast-specific Cre recombinase transgene driven by the CYP19 promoter to knock down placental mTOR, Akhaphong and co-workers previously reported decreased fetal weight in females but not in male pups [45]. However, the data in this previous study is difficult to interpret given that a conditional gene-targeting approach was not used, making it difficult to differentiate between the effect on placental development and function, neither inhibition of placental the signaling nor specificity of the mTOR knockdown to the trophoblast only was confirmed, and placental function was not assessed [45].

This study demonstrates that mTOR regulates the trafficking of LAT1 to the fetal facing basal plasma membrane in PHT cells. System L amino acid transporters facilitate the efflux of essential amino acids from the syncytiotrophoblast across the basal membrane into the fetal circulation [63]. *In vivo* stable isotope tracer studies indicate a decreased transfer of leucine from mother to fetus in cases of human FGR [64]. Reduced System L amino acid transport activity has been reported in BM isolated from the placentas of human FGR [11]. We and others have shown that placental mTOR signaling reduced in human FGR [12,29–32] and various animal models of FGR [33–41]. These observations support the model that placental mTOR inhibition is associated with decreased LAT1 transporter trafficking to the basal plasma membrane and reduced BM System L transporter activity in FGR placentas. Our findings have significance for our understanding of FGR development because fetal amino acid availability is crucial for growth [33].

Trophoblast mTOR KD will affect a number of downstream functions, including protein synthesis, autophagy, and mitochondrial respiration, in addition to nutrient transport. In this study, we aimed to investigate the functional relationship between trophoblast mTOR expression/activity, nutrient transport, and fetal growth. Specifically, we examined if trophoblast mTOR inhibition leads to a reduction in nutrient transport and fetal growth. This would support the idea that ‘rescuing’ placental mTOR signaling may represent a novel therapeutic strategy to prevent/treat abnormal fetal growth. In general support of this speculation, we have reported the effect of placenta-specific targeting of genes encoding for LAT1 AND SNAT2 amino acid transport isoforms regulated by mTOR (refs). Specifically, trophoblast-specific overexpression of *Lat1* led to increased nutrient transport and fetal growth [65] whereas trophoblast specific silencing of *Snat2* caused decreased nutrient transport and FGR [58] in mice, demonstrating cause and effect relationship between nutrient transport and fetal growth.

Our findings have clear translational significance. First, placental mTOR signaling is inhibited in human FGR [12,29–32] and increased in fetal overgrowth associated with maternal obesity and/or GDM [14,42–44,66]. Second, placental System L activity has been reported to be reduced in pregnancies complicated by FGR [11,64], associated with a decrease in fetal circulating concentrations of many essential amino acids [67,68]. In addition, SNAT2 (SLC38A2) protein expression and/or System A activity are decreased in term placenta of FGR or small for gestational age fetuses [9,10,69,70]. Maternal obesity, which increases the risk of fetal overgrowth, has been reported to be associated with increased placental amino acid transport capacity [14]. Specifically, System A activity, but not System L, was positively correlated with birth weight in a cohort of normal and obese women, and transplacental amino acid transport mediated by System A is increased in a mouse model of maternal obesity and fetal overgrowth [71]. Thus, the mechanistic link between inhibition of placental mTOR signaling, reduced placental amino acid transport activity, and FGR reported here provides novel insights into the causes of human FGR. One of the limitations of the current study is that we did not study the sex-specific effect of placental *Mtor* signaling on fetal growth and nutrient transport.

Recent findings suggest that the placenta fine-tunes the supply of maternal resources to the fetus in accordance with both the genetically determined fetal drive for growth and the maternal ability to supply the nutrients required for fetal growth [72]. In addition, Sferruzzi-Perri et al. [72] demonstrated in genetically modified mice that fetal signals play an important role in modulating placental function and fetal nutrient delivery. Furthermore, placental hormones such as chorionic somatomammotropin/IGF’s, play a significant role in fetal development, potentially by modifying maternal and fetal metabolism [73,74]. The contribution of the fetal and maternal hormone/signal/metabolism to placental phenotypes were not determined in the current study. Future work will determine the interrelationship between changes in the placental *Mtor* signaling and fetal and maternal hormone/signal/metabolism.

Because placental mTOR signaling responds to an array of maternal metabolic and nutritional signals and is a master regulator of placental function, including nutrient transport, mitochondrial function, and protein synthesis, we have proposed that placental mTOR signaling functions as a critical hub linking maternal nutrition and metabolism and utero-placental blood flow to placental function, fetal growth and developmental programming [27,75,76]. For example, when the maternal supply line is unable to support normal fetal growth due to maternal undernutrition or reduced utero-placental blood flow, placental mTOR signaling is inhibited resulting in down-regulation of multiple key placental functions, directly contributing to reduced fetal growth. We have speculated that this control system has developed due to the evolutionary pressure of starvation to protect the mother from demise at the cost of a smaller fetus. We also argue that this regulatory system responds to nutritional cures in the opposite direction, i.e., overnutrition in conditions such as maternal obesity and gestational diabetes, where placental mTOR signaling activation may contribute to fetal overgrowth in some cases. Thus, our current report establishing a direct mechanistic link between placental mTOR signaling and nutrient transport and fetal growth in mice, supports this model.

In the present study, we developed a novel method for achieving inducible trophoblast-specific *Mtor* knockdown *in vivo* in mice, allowing us to generate novel mechanistic information on regulating placental function and fetal growth. We demonstrate that the inhibition of trophoblast mTOR signaling in late pregnancy is mechanistically linked to decreased placental nutrient transport and reduced fetal growth. Currently, no specific intervention strategies are available that can be used clinically to treat FGR and fetal overgrowth. We speculate that trophoblast-specific targeting of mTOR signaling represents a promising avenue to improve outcomes in pregnancies complicated by abnormal fetal growth. This could be made possible by recent developments in nanotechnology, which have provided an innovative approach for drug delivery to or gene targeting of the placenta [77].

**Clinical perspectives**

- Fetal growth restriction (FGR) is an obstetric disease that severely compromises perinatal survival and long-term health of the infant. The biological mechanisms underlying FGR are unknown, preventing us from developing treatments.
- We show that PiggyBac transposase-mediated inducible trophoblast-specific knockdown of *Mtor* decreases trophoblast nutrient transport and fetal growth, demonstrating a mechanistic relationship between low trophoblast *Mtor* expression and FGR *in vivo*. We also show that silencing of *MTOR* in cultured primary human trophoblast cells lowered the amino acid transport activity and decreased the membrane trafficking of amino acid transporter isoforms.
- Our findings support the concept that trophoblast *MTOR* inhibition underpins human FGR and suggest that interventions that augment trophoblast mTOR signaling could improve fetal growth and perinatal outcomes in severe FGR.

## Supplementary material

Online supplementary figure 1

Online supplementary figure 2

Online supplementary figure 3

Online supplementary figure 4

Online supplementary figure 5

Online supplementary figure 6

Online supplementary figure 7

Online supplementary figure 8

Online supplementary figure 9

Online supplementary figure 10

Online supplementary figure 11

Online supplementary figure 12

## Data Availability

All supporting data and associated protocols for this manuscript are available upon request.

## Open Access

This article has been published open access under our Subscribe to Open programme, made possible through the support of our subscribing institutions, learn more here: https://portlandpress.com/pages/open_access_options_and_prices#conditional

## Abbreviations

FGR: fetal growth restriction
UTR: untranslated region
mTOR: mechanistic target of the rapamycin
te-PNI: transposase-enhanced pronuclear injection
