# Supplementary figures and images for: PiggyBac transposase-mediated inducible trophoblast-specific knockdown of Mtor decreases placental nutrient transport and fetal growth
Source: Clin Sci (Lond). 2025 Jul 31;139(14):825–45. doi: 10.1042/CS20243293 (PMC12409993; doi:10.1042/CS20243293)

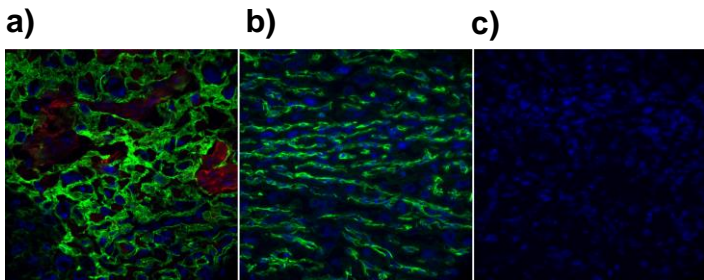

**Supplemental Figure 1**

Supplement: Online supplementary figure 1 [file CS-139-14-CS20243293-s001.pdf]

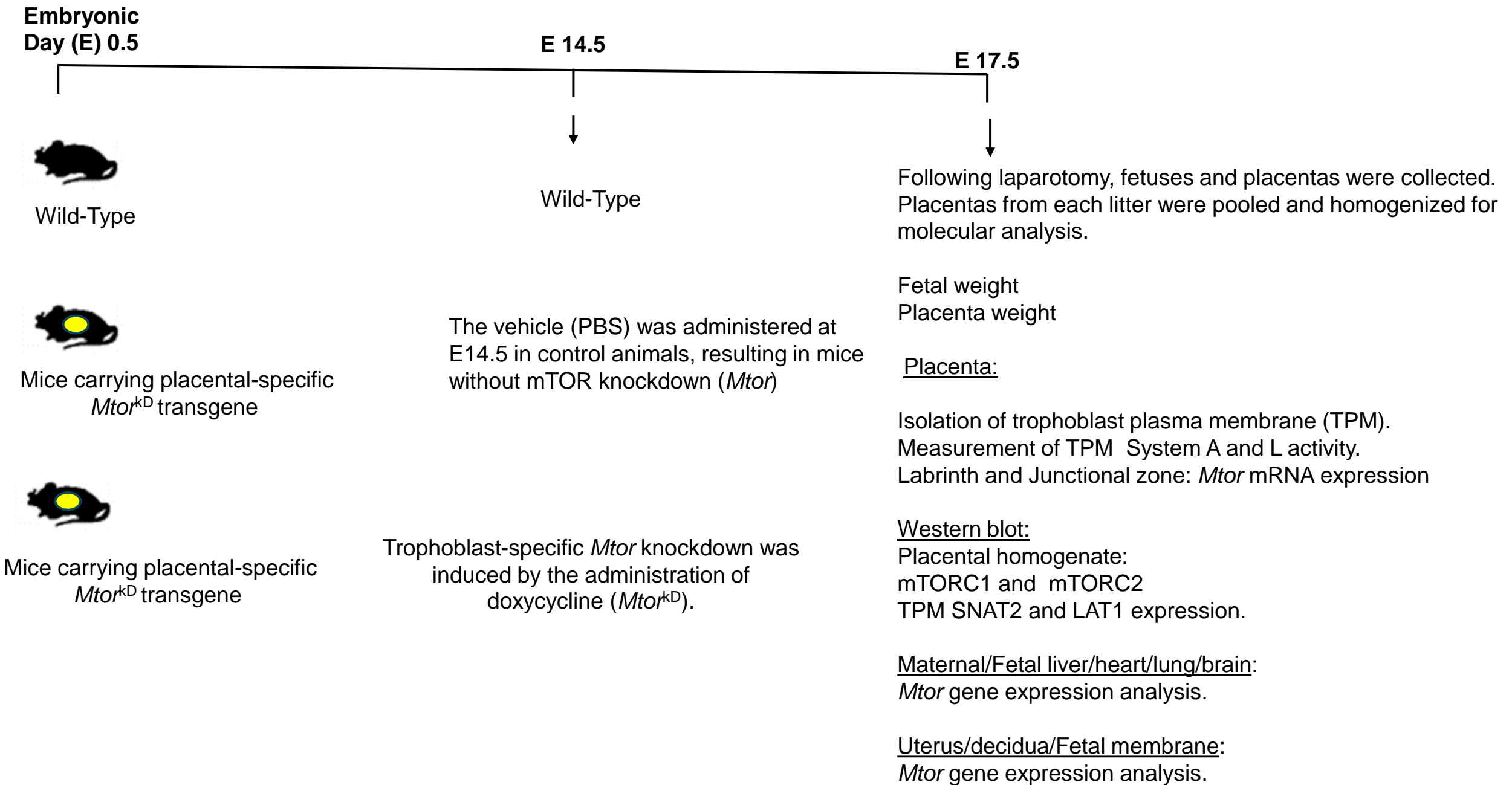

**Supplemental Figure 2**

Supplement: Online supplementary figure 2 [file CS-139-14-CS20243293-s002.pdf]

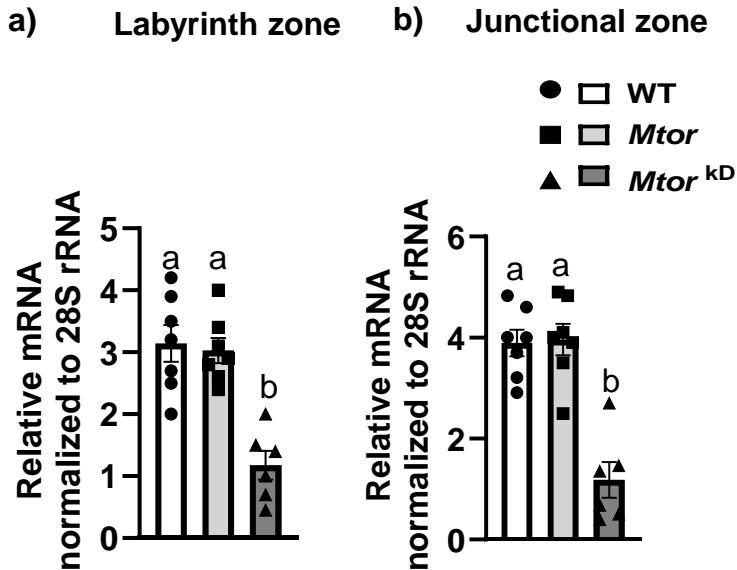

Supplemental Figure 3

Supplement: Online supplementary figure 3 [file CS-139-14-CS20243293-s003.pdf]

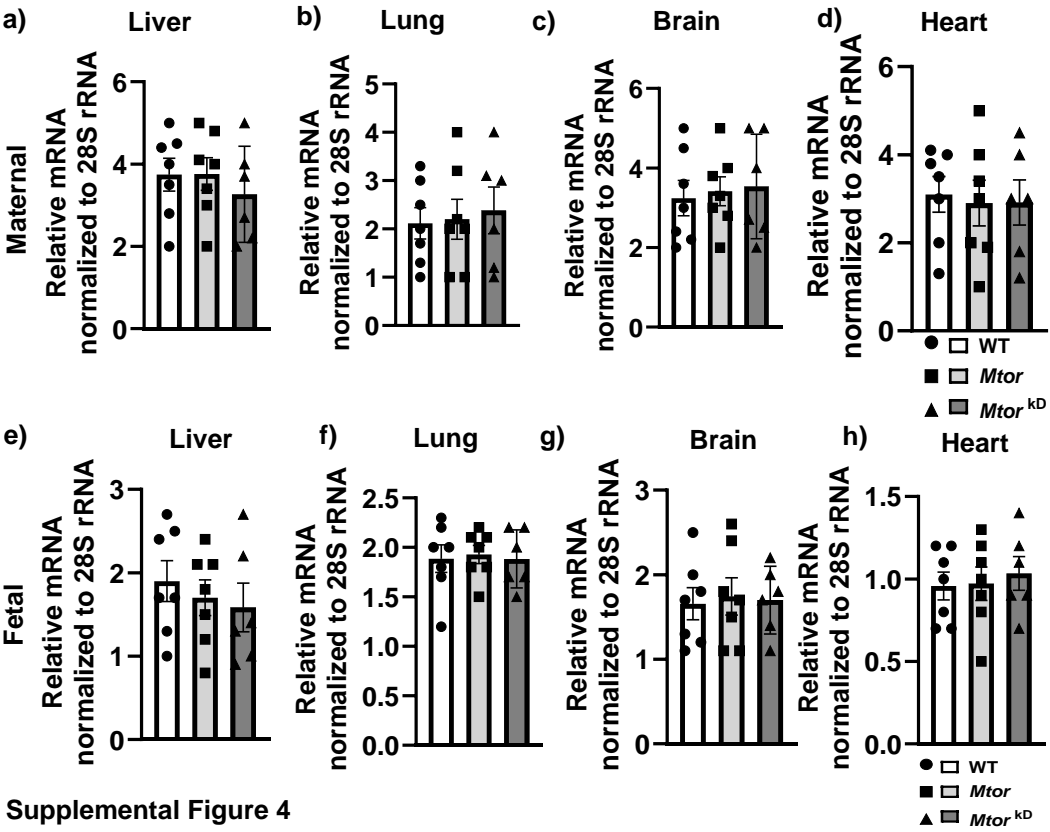

Supplemental Figure 4

Supplement: Online supplementary figure 4 [file CS-139-14-CS20243293-s004.pdf]

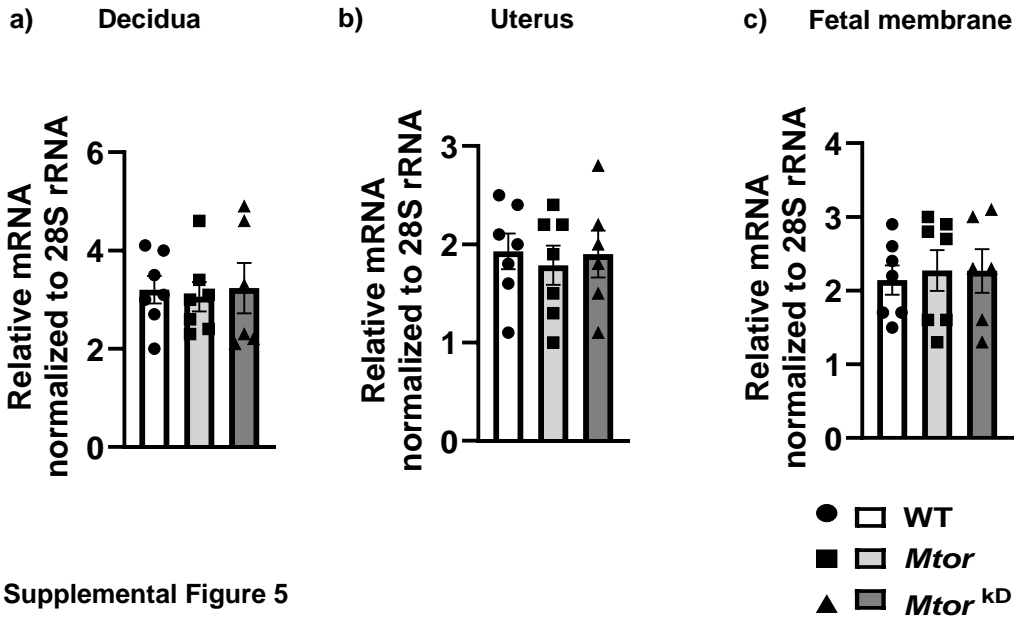

Supplemental Figure 5

Supplement: Online supplementary figure 5 [file CS-139-14-CS20243293-s005.pdf]

a)

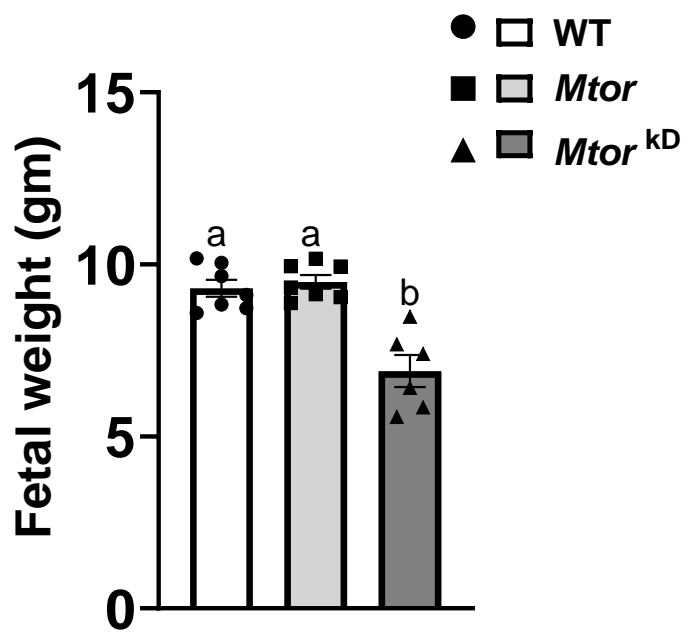

b)

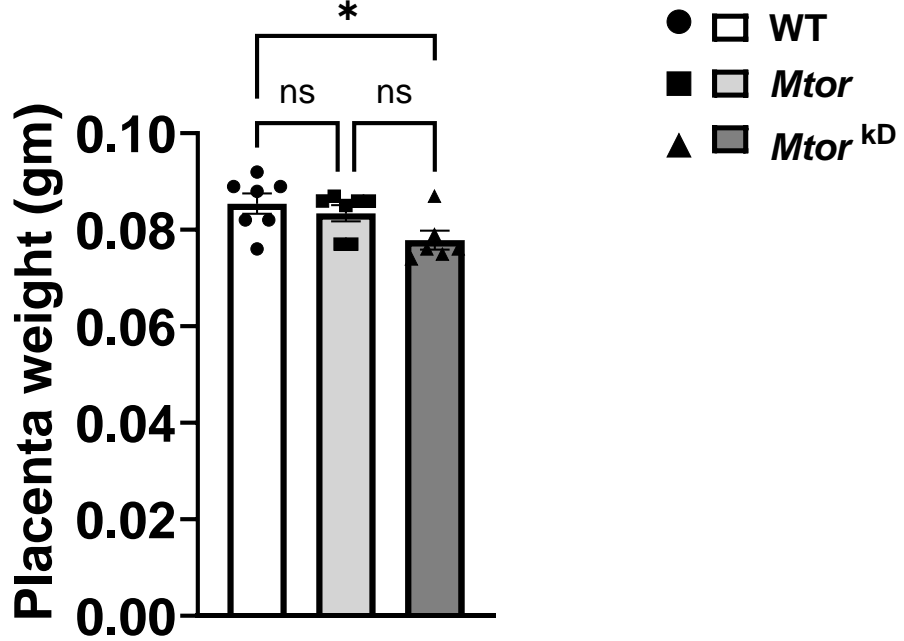

c)

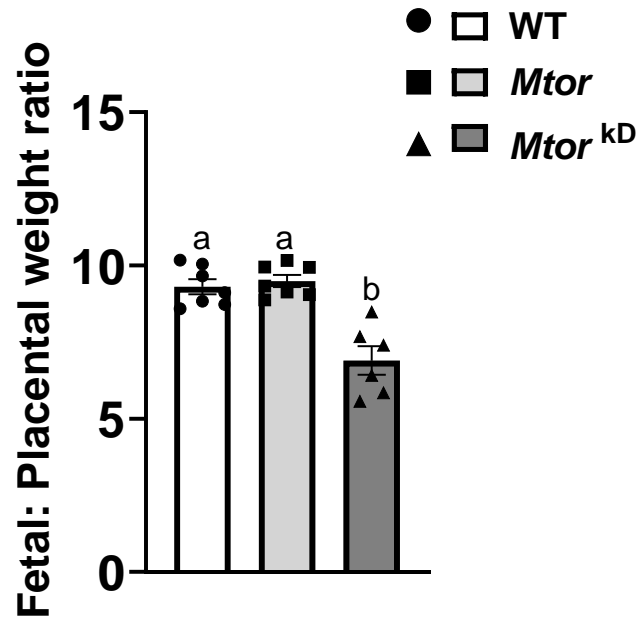

Supplement: Online supplementary figure 6 [file CS-139-14-CS20243293-s006.pdf]

a)

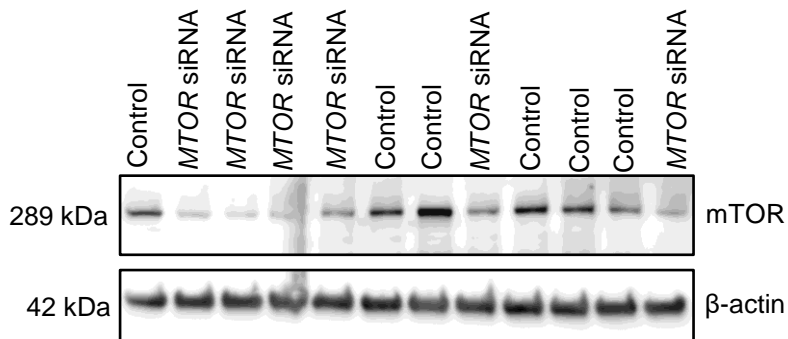

b)

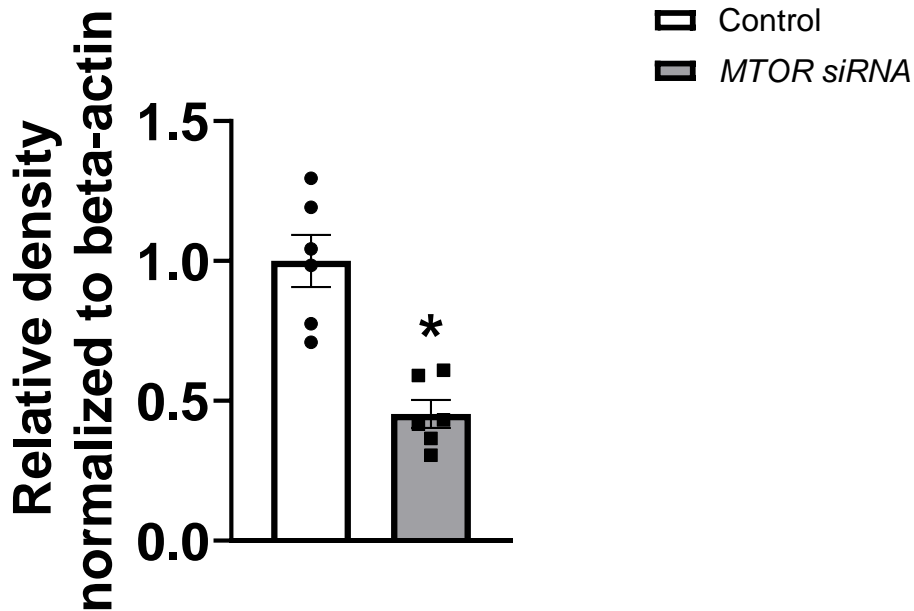

Supplement: Online supplementary figure 7 [file CS-139-14-CS20243293-s007.pdf]

a)

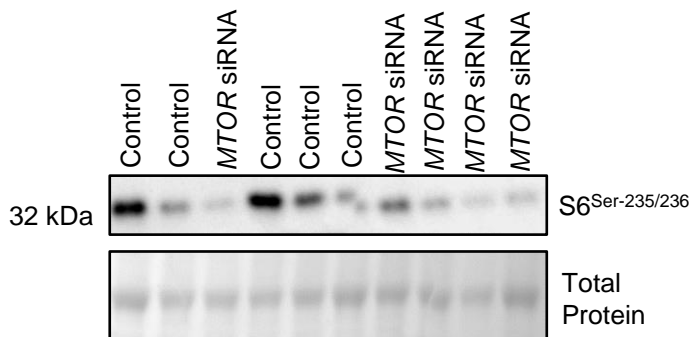

b)

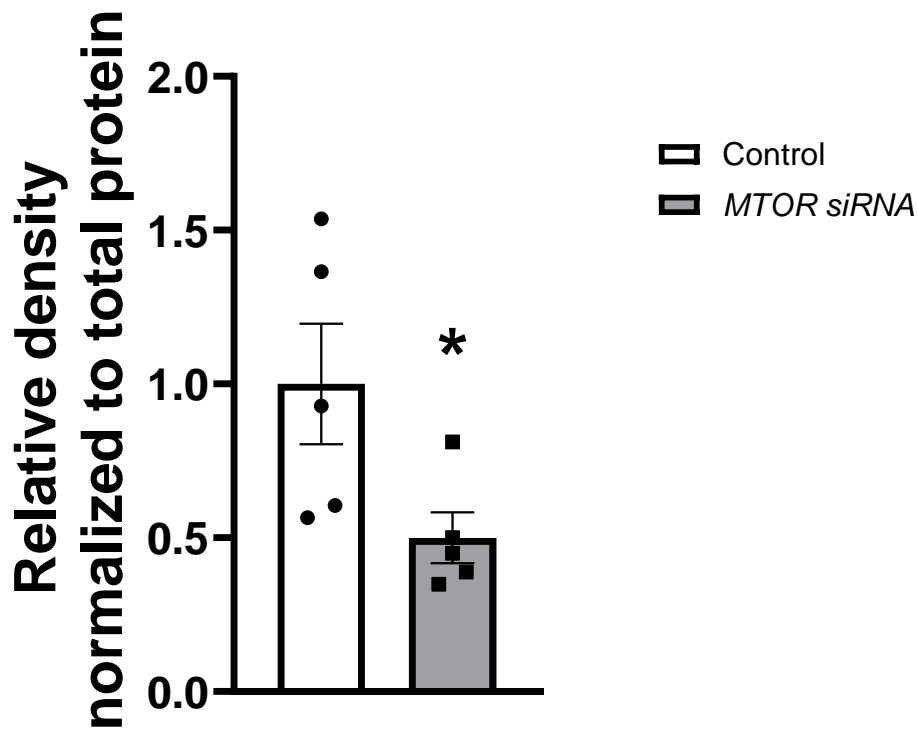

Supplement: Online supplementary figure 8 [file CS-139-14-CS20243293-s008.pdf]

a)

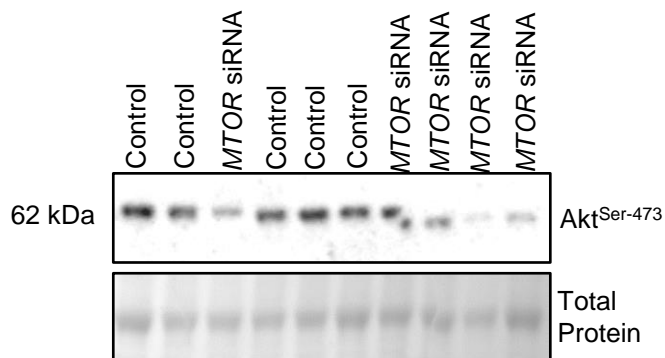

b)

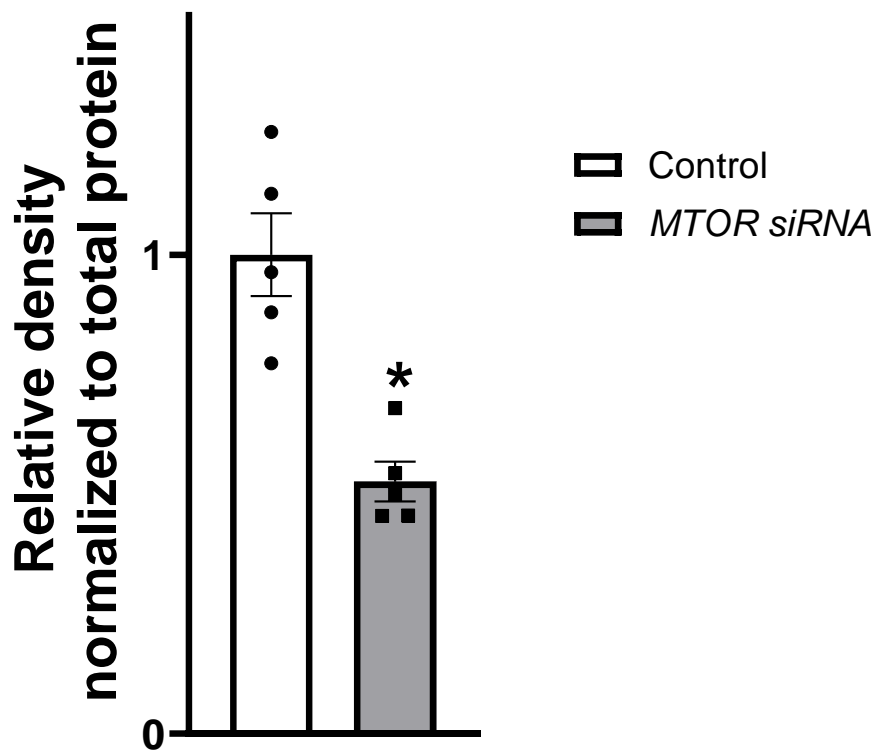

Supplement: Online supplementary figure 9 [file CS-139-14-CS20243293-s009.pdf]

a)

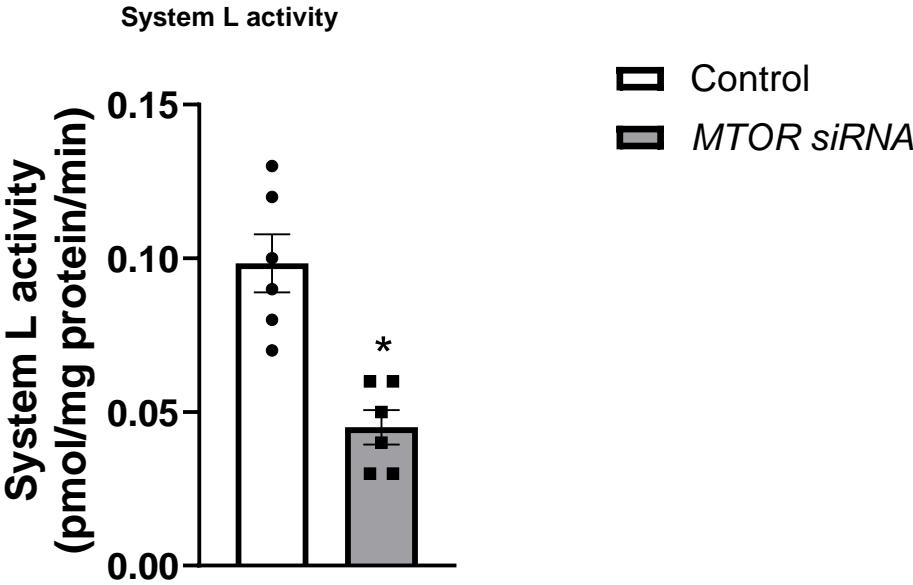

b)

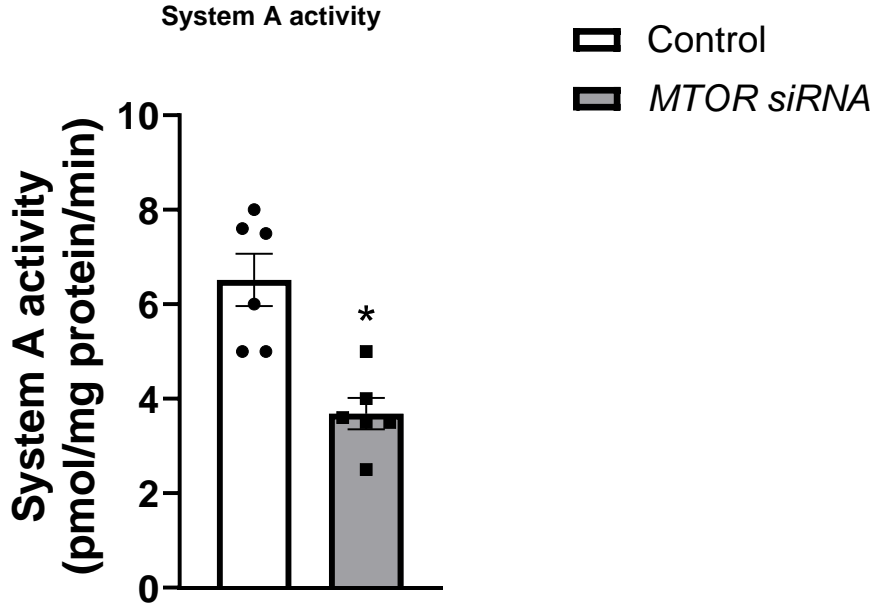

Supplement: Online supplementary figure 10 [file CS-139-14-CS20243293-s010.pdf]

a)

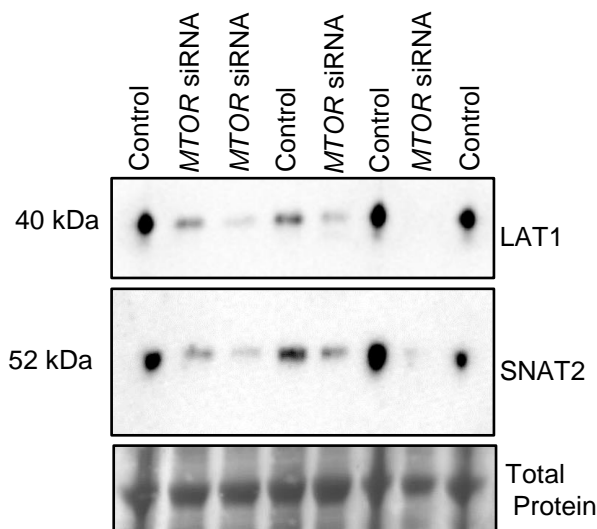

b)

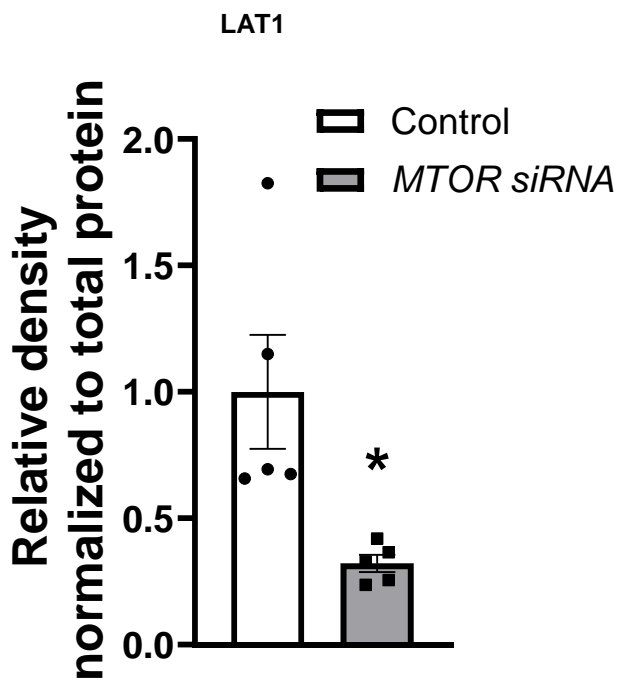

c)

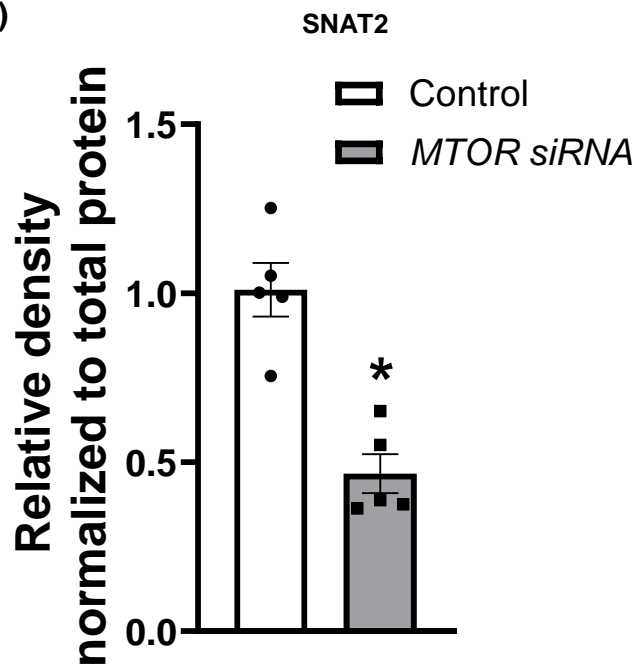

Supplement: Online supplementary figure 11 [file CS-139-14-CS20243293-s011.pdf]

a)

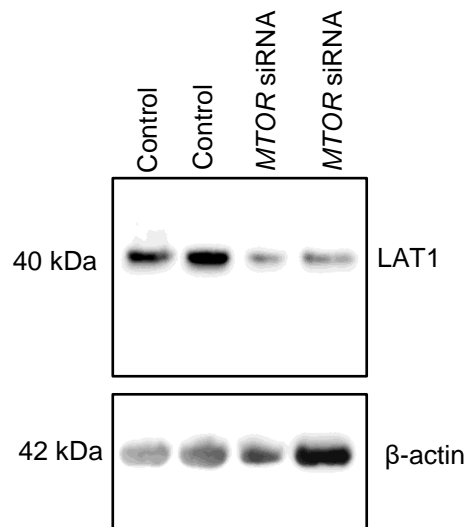

b)

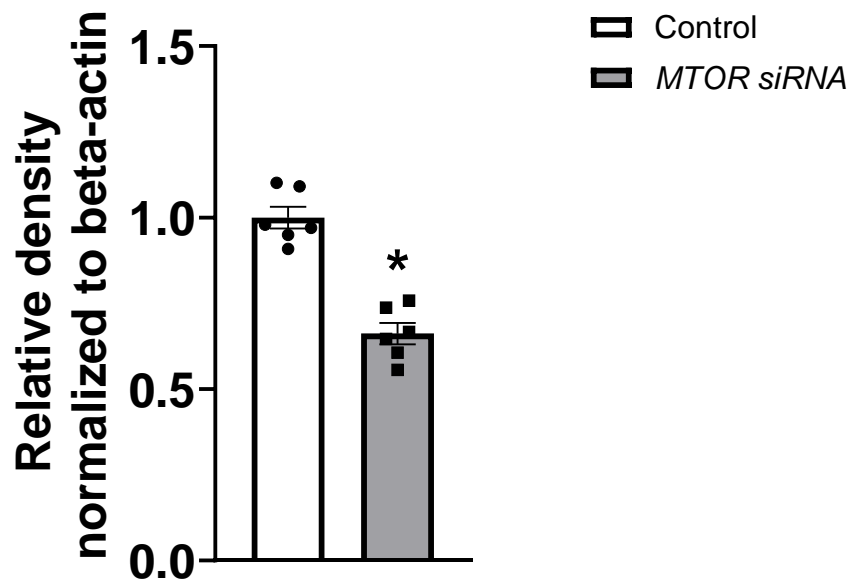

Supplement: Online supplementary figure 12 [file CS-139-14-CS20243293-s012.pdf]
